# Using Fuzzy C-Means Clustering to Determine First Arrival of Microseismic Recordings

**DOI:** 10.3390/s24051682

**Published:** 2024-03-05

**Authors:** Xiangyun Zhao, Haihang Chen, Binhong Li, Zhen Yang, Huailiang Li

**Affiliations:** 1 Key Laboratory of Earth Exploration and Information Technology, Ministry of Education, Chengdu University of Technology, Chengdu 610059, China; zxy@stu.cdut.edu.cn (X.Z.); yangzhen30@stu.cdut.edu.cn (Z.Y.); libinhon2024@163.com (B.L.); 2 College of Earth Science, Chengdu University of Technology, Chengdu 610059, China; 3 State Key Laboratory of Geohazard Prevention and Geoenvironment Protection, Chengdu University of Technology, Chengdu 610059, China

**Keywords:** microseismic data, first-arrival picking, fuzzy c-means clustering, machine learning

## Abstract

Accurate and automatic first-arrival picking is one of the most crucial steps in microseismic monitoring. We propose a method based on fuzzy c-means clustering (FCC) to accurately divide microseismic data into useful waveform and noise sections. The microseismic recordings’ polarization linearity, variance, and energy are employed as inputs for the fuzzy clustering algorithm. The FCC produces a membership degree matrix that calculates the membership degree of each feature belonging to each cluster. The data section with the higher membership degree is identified as the useful waveform section, whose first point is determined as the first arrival. The extracted polarization linearity improves the classification performance of the fuzzy clustering algorithm, thereby enhancing the accuracy of first-arrival picking. Comparison tests using synthetic data with different signal-to-noise ratios (SNRs) demonstrate that the proposed method ensures that 94.3% of the first arrivals picked have an error within 2 ms when *SNR* = −5 dB, surpassing the residual U-Net, Akaike information criterion, and short/long time average ratio approaches. In addition, the proposed method achieves a picking accuracy of over 95% in the real dataset tests without requiring labelled data.

## 1. Introduction

Microseismic events can be triggered by various engineering activities such as shale gas extraction [1], tunnel engineering [2], rock slope stability monitoring [3], hydropower station construction [4], and deep mining [5]. First-arrival picking is one of the most crucial steps in microseismic monitoring. Although manual picking has exhibited high reliability and yielded favorable outcomes in the past, it becomes nearly impossible when dealing with large amounts of data or data that are poor quality. Nowadays, many underground engineering activities generate numerous microseismic data with varying quality. Consequently, automatic approaches for first-arrival picking have emerged as the preferred choice in practical applications [6].

Over the past few decades, researchers have proposed numerous well-established methods for first-arrival picking, such as the short/long time average ratio (STA/LTA) [7], Akaike information criterion (AIC) [8] and various improved approaches [9,10], wavelet denoising and its improved forms [11,12], PAI-S/K based on skewness and kurtosis [13], STK/LTK based on the long- and short-time window kurtosis ratio [14], the Markov optimal decision process [15], waveform similarity-based approaches [16], and improved multi-channel cross-correlation [17]. The STA/LTA offers the advantages of simplicity and computational efficiency in first-arrival picking. However, the accuracy of the STA/LTA is influenced by inappropriate window lengths and triggering thresholds [18,19]. The AIC picker exhibits several advantages in single-phase first-arrival picking, including its ease of implementation, fast computation speed, and high accuracy [20,21]. However, in multi-phase first-arrival picking, the AIC picker is prone to confusion during multiple phases of picking [22]. These traditional approaches rely heavily on the experience of human experts and involve manual intervention and subjective decision making based on professional knowledge and experience [23,24]. Moreover, the aforementioned methods are sensitive to noise and require the use of strong denoising techniques to achieve good results [25,26]. Motivated by the observations above, a more accurate and robust automatic first-arrival picking method needs to be developed with reduced human bias and improved noise tolerance.

Deep learning, a powerful artificial intelligence technique, has found extensive application in various domains owing to its ability to extract rich features from data. For automatic first-arrival picking, deep learning has emerged as a prominent alternative to conventional approaches, as it can handle large datasets without requiring professional knowledge [27]. Particularly in recent years, several effective deep learning approaches have been presented for first-arrival picking, such as the combination of a regression convolutional neural network (CNN) and AIC [21], the parallel dual task network (PDTN) [28], CNN-based methods [29,30], feature pyramid networks (FPNs) [31], acoustic emission AEnet [32], CapsNet [33,34], PhaseNet [35], PickNet [36], the pixel-level network [37], U-net [38], and improved picking approaches using deep learning [39,40]. These approaches have demonstrated superior performance compared to traditional methods. However, the effectiveness of these algorithms is heavily influenced by the quantity and quality of labelled training datasets. Consequently, In scenarios where large labelled datasets are unavailable or collecting them is expensive, unsupervised machine learning methods that learn patterns directly from the data based on sample similarity may present the optimal choice for picking the first arrival in microseismic recordings [41,42]. Clustering is an effective unsupervised learning tool employed to classify unlabeled seismic data into distinct clusters [43]. In recent years, numerous effective clustering methods have been developed for first-arrival picking; fuzzy clustering methods such as fuzzy c-means and its variants [9,44,45] have been successfully utilized, and Zhu et al. [46] achieved good results by using three time-domain features (power, mean, and variance) from one-component seismic data as conditions for fuzzy clustering, which also provided us with valuable insights. Additionally, k-means and its improved forms [22,47] have shown promising results for phase picking. These approaches offer the advantage of not requiring large quantities of labelled data or extensive model training, thus providing greater flexibility and lower requirements in terms of usage conditions.

In this sense, although clustering-based approaches are relatively old, these methods do not require massive amounts of labelled datasets or model training. To achieve accurate and rapid first-arrival picking for microseismic recordings, we developed a straightforward picking method employing fuzzy c-means clustering with a limited amount of high-quality human-labelled data available, which transforms the arrival-time picking task into clustering useful waveform and noise sections for a raw microseismic waveform.

## 2. Methods

### 2.1. Fuzzy C-Means Clustering

For the target dataset $X=[x_{1},x_{2},$…$,x_{n}]$, the fuzzy c-means clustering (FCC) method minimizes the objective function value $J_{fcm}$ by iteratively calculating the cluster center $c_{j}$ and the membership degree $u_{ij}$. The standard objective function is given as follows:

(1) $$J_{fcm}=\sum_{i=1}^{n}\sum_{j=1}^{c}u_{ij}^{m}{d_{ij}}^{2}$$

where *n* is the number of data points, *c* is the number of clusters (c≥2), *m* is the fuzziness index (m>1) that represents the level of cluster fuzziness, $u_{ij}$ is the membership of data point $x_{i}$ belonging to cluster *j*, which ranges from 0 to 1, and $d_{ij}=\left|\right|x_{i}-c_{j}\left|\right|$ is the euclidean spatial distance between data point $x_{i}$ and cluster center $c_{j}$; *i* is the *i*th sample and *j* is the *j*th cluster. The closer $u_{ij}$ is to 1, the more likely it is that the data point $x_{i}$ belongs to cluster *j*. The definition of restriction $u_{ij}$ is given as:

(2) $$\sum_{j=1}^{c}u_{ij}=1,i=1,2,\ldots ,n$$

In which, a smaller value of $J_{fcm}$ indicates better classification performance. The problem described in Equation (1) involves finding the optimal values for $c_{j}$ and $u_{ij}$. The clustering center and membership degree are updated by the Equations (3) and (4), respectively.

(3) $$c_{j}=\frac{\sum_{i=1}^{n}u_{ij}^{m}x_{i}}{\sum_{i=1}^{n}u_{ij}^{m}}$$

(4) $$u_{ij}=\frac{1}{\sum_{k=1}^{c}{\left(\frac{∥x_{i}-c_{j}∥}{∥x_{i}-c_{k}∥}\right)}^{2/(m-1)}}$$

(5) $$∥J_{fcm}\left(T+1\right)-J_{fcm}\left(T\right)∥\leq \xi$$

where *T* is the iteration number and ξ is a small enough positive number. During the iteration process, $c_{k}(k=1,2,$…c) represents the cluster center for the *k*th cluster, and $c_{j}$ and $u_{ij}$ are continually updated until the convergence of Equation (5) is completed.

### 2.2. Features Extraction for Fuzzy Clustering

Feature factors are employed as the fuzzy local similarity measures for the objective function. The purpose of selecting feature factors is to assign the data points with high similarity to one cluster and those with low similarity to another cluster. In this paper, we introduce power, variance, and polarization linearity as feature factors.

Assuming that $X=[x_{i},y_{i},z_{i},i=1,2,$…,n] represents the 3C microseismic recording of *n* data points, for each continuous sequence of *q* data points (empirically, q=21 in this study), we calculate their power, variance, and polarization linearity as distinctive feature factors for the center point. Power $P_{i}$ and variance $S_{i}$ are defined as follows:

(6) $$P_{i}=\sum_{i-(q-1)/2}^{i+(q-1)/2}x_{i}^{2}$$

(7) $$S_{i}=\sqrt{\frac{1}{q}\sum_{i-(q-1)/2}^{i+(q-1)/2}{\left(x_{i}-{\bar{x}}_{i}\right)}^{2}}$$

where ${\bar{x}}_{i}$ is the mean value of $x_{i}$ and *q* represents the window length of the cluster analysis. As for the polarization linearity feature $L_{i}$, we firstly intercept the 3C microseismic recording with window length *q*, and construct the covariance matrix *M* using Equation (8) [48].

(8) $$M_{i}=\left[\begin{array}{ccc} var(x) & cov(x,y) & cov(x,z)\\ cov(y,x) & var(y) & cov(y,z)\\ cov(z,x) & cov(z,y) & var(z) \end{array}\right]$$

where *x*, *y*, and *z* represent two horizontal and a single vertical component data, respectively. cov(x,y) and var(x) are the covariances between *x* and *y*, and *x* and *x*, respectively. Next, we use Equation (9) to calculate the eigenvalues λ and eigenvectors V of *M*, and the eigenvalues $\lambda_{1}>\lambda_{2}>\lambda_{3}$.

(9) $$M_{i}=V\left[\begin{array}{ccc} \lambda_{1} & 0 & 0\\ 0 & \lambda_{2} & 0\\ 0 & 0 & \lambda_{3} \end{array}\right]V^{T}$$

Finally, the polarization linearity $L_{i}$ is calculated as follows [49]:

(10) $$L_{i}=\frac{{\left(\lambda_{1}-\lambda_{2}\right)}^{2}+{\left(\lambda_{1}-\lambda_{3}\right)}^{2}+{\left(\lambda_{2}-\lambda_{3}\right)}^{2}}{2{\left(\lambda_{1}+\lambda_{2}+\lambda_{3}\right)}^{2}}$$

### 2.3. Fuzzy C-Means Clustering for First-Arrival Picking

**Figure 1:**
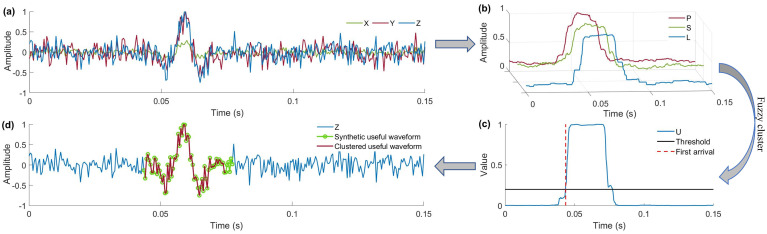
Useful waveform and noise clustering process of the proposed method. (**a**) A synthetic noisy three-component (3C) microseismic recording using Rick wavelet with a duration of 0.15 s, a principal frequency of 100 Hz, a sample rate of 2 kHz, and an *SNR* of −2 dB. (**b**) *P*, *S*, and *L* features of microseismic recording represent the energy, variance, and polarization linearity, respectively. (**c**) The membership degree of the useful synthetic waveform; the black line is the classifier threshold ξ, red dashed line is the first arrival identified by the proposed method. (**d**) The clustering result; the blue line is the noisy Z component of the 3C microseismic recording, the green dot is the synthetic, useful waveform, and the red line is the clustered useful waveform identified by the proposed method.

As an unsupervised machine learning method, the FCC method aims to divide microseismic recordings into useful waveforms and noise, and the first point of the useful waveform is identified as the first arrival of the microseismic event. The entire picking procedure is shown in Figure 1. It can be observed from Figure 1c that there is a distinct first arrival when there is a significant fluctuation in the membership degree *U* of the useful waveform section. Using this criterion, the essence of the first-arrival picking is transformed into the clustering operation of a given set of data points, and the accuracy of the first-arrival picking is directly related to the accuracy of the clustering.

In this study, we set the cluster number as c=2 and fuzziness index as m=2, constructing feature matrix dataset $E_{i}$ which is defined by Equation (11) based on the features calculated using Equations (6), (7) and (10). The $d_{ij}$ is the Euclidean distance between the feature matrix dataset $E_{i}$ and the cluster center $c_{j}$.

(11) $$E_{i}=\left[P_{i},S_{i},L_{i}\right]$$

(12) $$d_{ij}=\sqrt{{\left(P_{i}-C_{Pj}\right)}^{2}+{\left(S_{i}-C_{Sj}\right)}^{2}+{\left(L_{i}-C_{Lj}\right)}^{2}}$$

where $C_{Pj}$, $C_{Sj}$, and $C_{Lj}$ are the *j*th cluster centers of *P*, *S*, and *L*, respectively.

The dataset $E_{i}$ is used as the input for FCC to obtain the final membership matrix $u_{ij}$, which contains membership degrees of useful waveforms and noise. The membership degrees *U* of useful waveform clusters are obtained from Equation (13). We define data points with U>σ (with a threshold σ=0.4) as useful waveform sections, while those below the threshold are considered as noise sections. Consequently, the first point in the useful waveform is determined as the first arrival.

(13) $$U=\{u_{ik}|i\in \left[1,n\right],k=\underset{j}{\arg\min}\sum_{i=1}^{n}u_{ij}\}$$

where *n* is the number of data points, *i* is the *i*th sample, *j* is the *j*th cluster, $u_{ij}$ is the membership of data point $x_{i}$ belonging to cluster *j*. We consider the cluster *j* that satisfies the conditions $\min_{j}\sum_{i=1}^{n}u_{ij}$ as the useful waveform cluster *k*; $u_{ik}$ is the membership of data point $x_{i}$ belonging to useful waveform cluster *k*.

In addition, updating the membership degree involves a process that highlights the similarity of feature factors. Compared with the method proposed by [9], we give a suggested window length (as shown in Figure 6) to extract the corresponding features. By incorporating the more robust feature *L*, the FCC method greatly enhances signal characteristics while reducing the impact of noise. Consequently, effective clustering is achieved on the calculated *U*, significantly improving the accuracy of first-arrival picking.

## 3. Tests and Results

### 3.1. Synthetic Data Test

**Figure 2:**
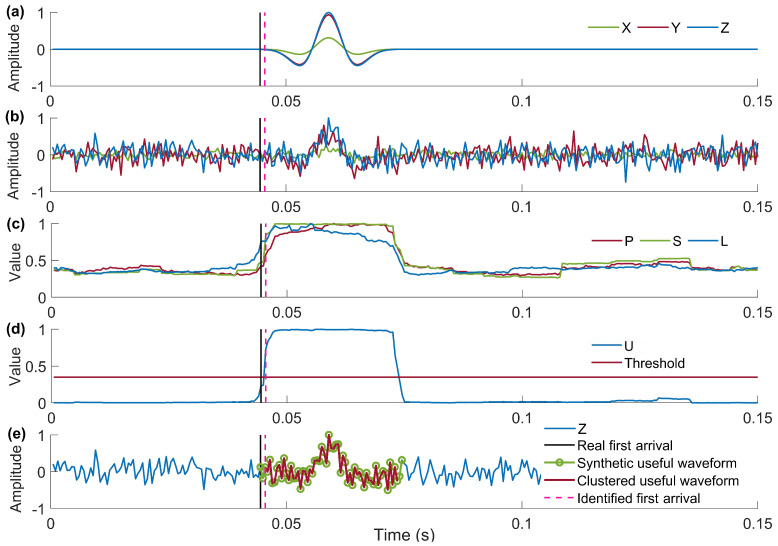
First-arrival picking result of the proposed method at *SNR* = −5 dB. (**a**) A synthetic pure 3C microseismic recording using Rick wavelet with a duration of 0.15 s, a principal frequency of 100 Hz, and a sample rate of 2 kHz. (**b**) The corresponding noisy microseismic recording with *SNR* = −5 dB, and the green, red, and blue lines are the same as (**a**). (**c**) Features *P*, *S*, and *L* represent the energy, variance, and polarization linearity extracted from (**b**). (**d**) The membership degree curve and threshold of the synthetic useful waveform cluster. (**e**) Results of first-arrival picking and waveform clusters using the proposed method.

Figure 2 shows the first-arrival picking result of the proposed method at *SNR* = −5 dB, and it can be seen that the proposed method has a picking error of 3.2 ms. To further validate the reliability of the proposed method and quantify its picking errors, we performed 1000 repeated tests using synthetic data, and the test results are shown in Table 1. The *SNR* is defined as

(14) $$SNR=10\log_{10}\frac{\sum_{t}{\left|s\left(t\right)\right|}^{2}}{\sum_{t}{\left|x\left(t\right)-s\left(t\right)\right|}^{2}}$$

where s(t) is the raw clean signal, and x(t) is the noisy signal.

**Figure 3:**
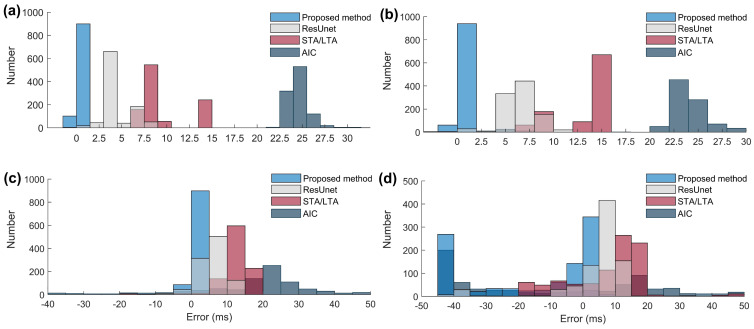
The distribution of the first-arrival picking errors for different methods. (**a**–**d**) represent the first-arrival picking errors with the SNRs of 5 dB, 0 dB, −5 dB, and −10 dB, respectively.

As can be seen from Table 1, when *SNR* = −5 dB, 94.2% of the picked first arrivals have an error within 2 ms, which confirms that the proposed method can achieve a reliable first-arrival estimation. Moreover, even when *SNR* = −8 dB, 63.7% of the picked first arrivals have an error within 2 ms. To further demonstrate the effectiveness of the proposed method, we compared it with traditional approaches such as STA/LTA and AIC, as well as the Residual U-Net (ResUnet). The error statistics for the test results, based on the data presented in Table 1, are shown in Figure 3.

It can be observed from Figure 3a that the presented method exhibits the highest accuracy at *SNR* = 5 dB, with the majority of errors being within 2 ms. The ResUnet performs relatively well, with errors primarily within 5 ms. In contrast, the STA/LTA shows errors within 10 ms, while the AIC method performs the least favorably, with errors around 25 ms, potentially due to its tendency to pick at the waveform’s tail. In Figure 3b, this trend remains consistent at *SNR* = 0 dB. As the *SNR* decreases, all approaches experience an increase in picking errors. However, at *SNR* = −10 dB, the error variation in our proposed method is significantly higher than that in other approaches’ methods, making it unreliable for data characterized by an extremely low *SNR*.

### 3.2. Test of Real Data

**Figure 4:**
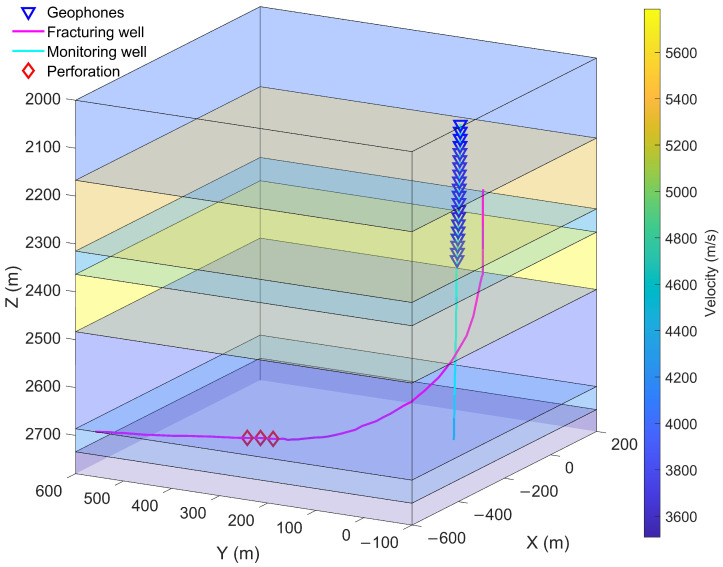
Downhole microseismic acquisition system in the work area. We employ 20 3C high-sensitivity moving-coil geophones with 1.0 Hz in the vertical monitoring well, the trace space is 15 m, and the sampling frequency is 2 kHz. The depth range is from 2515 m to 2800 m, The perforation depth is about 2730 m, the maximum depth of the geophone is 2405 m, the distance from the perforation to the monitoring well is about 330 m, and the wellhead of the monitoring well is approximately 50 m away from the fracturing well.

**Figure 5:**
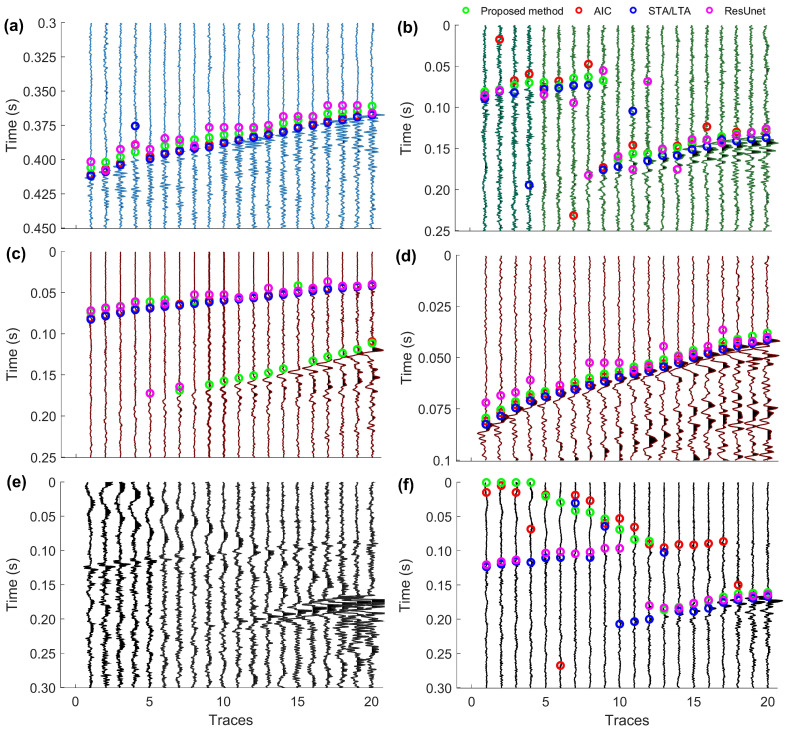
Comparison of the first-arrival picking results of the different approaches. (**a**) First-arrival picking results of the high *SNR* microseismic recordings. (**b**) First-arrival picking results of the low *SNR* microseismic recordings. (**c**) First-arrival picking results of a set of microseismic recordings containing P-waves and S-waves with high *SNR*, and (**d**) is the first-arrival picking results of the intercepted P-wave from (**c**). (**e**) A set of microseismic recordings including P-waves, S-waves, and interference waves with low *SNR*, and (**f**) is the first-arrival picking results of (**e**).

To confirm the reliability of the proposed method, we conducted comparison tests using real 3C microseismic recordings selected from a downhole hydraulic fracturing microseismic monitoring, and the acquisition system is shown in Figure 4. Several sets of continuous microseismic data with different SNRs and distinct first-arrival distributions were selected for testing. The results of the first-arrival picking are exhibited in Figure 5.

As shown in Figure 5a, all approaches achieve reliable first-arrival picking for microseismic recordings with high SNRs. The error of the ResUnet is slightly higher than those of other methods. Figure 5b shows a set of microseismic recordings with low SNRs, where the *SNR* gradually increases from left to right. It can be seen that these methods are ineffective when the *SNR* is too low (1–10 traces). However, within the 10–20 traces range, the proposed method demonstrates more stability than the other methods. In Figure 5c, we present a microseismic dataset characterized by a relatively high *SNR* and the presence of both P-waves and S-waves. The first-arrival picking results indicate that our method has a preference for identifying S-waves. Figure 5d only exhibits the picking results for the P-waves extracted from Figure 5c. From the obtained results, the proposed method demonstrates superior stability. Figure 5e displays a set of microseismic recordings with low SNRs, multiple phases, and significant interference waves. In Figure 5f, the ResUnet method demonstrates a superior performance in accurately picking the onsets of microseismic signals while avoiding interference waves. This success can be attributed to the labelled dataset, for which substantial preparatory work is required for this method.

## 4. Discussion

**Figure 6:**
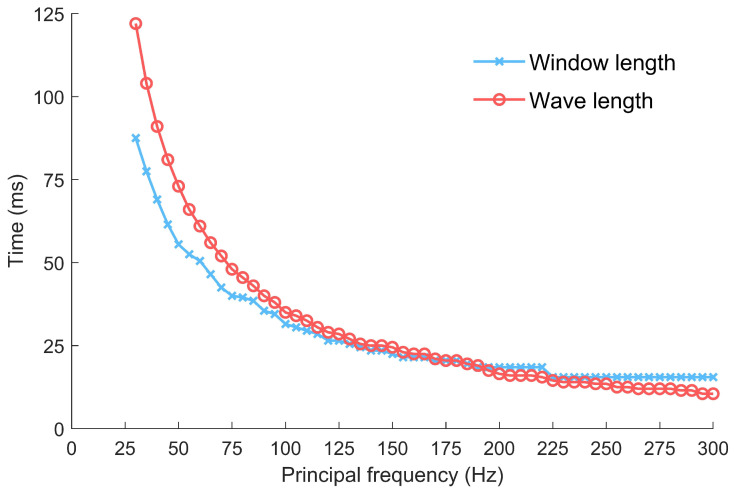
Interdependency among the principal frequency, window length, and wavelength.

Considering the previous test results and the calculation cost (the time consumptions of the proposed method, ResUnet, AIC, and STA/LTA are 0.016496 s, 2.826956 s, 0.004601 s, and 0.004305 s, respectively), the proposed method offers several advantages, such as speed, accuracy, and stability. However, the challenges lie in setting the sliding window length and cluster threshold. Automating this process would significantly enhance the practicality of the proposed method. Extensive tests have shown that the window length used in the proposed method can directly influence the feature curve and further indirectly affect the membership degree of the signals in fuzzy clustering. Conversely, the threshold σ of the cluster has a minimal impact on clustering results; as long as it is set to σ≥0.2, the difference in the clustering results is negligible. Therefore, we chose a fixed threshold of 0.2 and paid more attention to the sliding window length. Since higher-frequency seismic wavelets have better temporal resolution and the amplitude can reach its maximum faster, the chosen window length needs to be shorter, otherwise it should be longer. We conducted a series of tests using microseismic recordings with different principal frequencies and added white Gaussian noise with *SNR* = −5 dB; the results are shown in Figure 6. It can be observed that the clustering analysis window length should align with the wavelength and be approximately inversely proportional to the principal frequency of the microseismic recording.

It is worth mentioning from Figure 3 that, while both the STA/LTA and ResUnet methods exhibit stability throughout the test, their accuracy is not optimal, especially considering the additional data and time requirements for ResUnet during the preparation phase.

Notably, Figure 5f reveals that, when the amplitudes of the microseismic signals and interference signals are comparable (1–12 traces), our method tends to pick the first arrivals of interference signals. This outcome is expected since the features employed in our method are predominantly based on the time domain. Even when microseismic signals are less influenced by interference signals (13–20 traces), the developed method still accurately picks the first arrivals of microseismic signals. Overall, the proposed method exhibits a higher and more stable accuracy compared to other approaches.

Differently to the FCC-based method proposed by [46], this paper’s improvement lies in using three-component seismic data and adopting three features which are distinct from those in the referenced article, including the polarization linearity feature *L*, which enhances the clustering effect. We fixed the threshold at 0.4 and streamlined the data processing workflow. Our approach offers higher automation of first-arrival picking without compromising accuracy and response speed than the pre-improvement stage. In addition, for the same synthetic data with an *SNR* of −5 dB, our approach increased the first-arrival picking accuracy from 95.2% to 97.4%.

## 5. Conclusions

We present a precise first-arrival picking algorithm for microseismic recordings based on fuzzy clustering. This approach transforms the first-arrival picking task into a challenge of clustering useful waveforms and noise while incorporating polarization linearity features to enhance clustering accuracy. Experimental results demonstrate that the proposed method exhibits stability at different SNRs for microseismic recordings, even achieving a picking accuracy of 63.7% at *SNR* = −8 dB. Moreover, compared with the ResUnet method, the proposed approach does not require a large amount of labelled data or model training, making it simpler to implement, saving time, improving efficiency, and offering greater flexibility. It provides a feasible solution for potential automatic microseismic monitoring.

## Figures and Tables

**Table 1:** Comparison of the picking errors in the proposed method at different SNRs.

*SNR* (dB)	Number of Signals	Error (10 ms)	Error (2 ms)
5	1000	1000	1000
0	1000	1000	999
−5	1000	974	943
−7	1000	872	794
−8	1000	743	637
−10	1000	506	383

## Data Availability

This code and data can be obtained from the Github repository, which is located at https://github.com/malegaga/First-arrival-picking, accessed on 7 January 2024.
